# The History and Physical in Cancer Care: A Primer for the Oncology Advanced Practitioner

**DOI:** 10.6004/jadpro.2014.5.4.3

**Published:** 2014-07-01

**Authors:** Margaret Quinn Rosenzweig, Diane Gardner, Brenda Griffith

**Affiliations:** From University of Pittsburgh School of Nursing, Pittsburgh, Pennsylvania; University of Pittsburgh Cancer Institute, Pittsburgh, Pennsylvania; Allen Interactive Media Consultants, Minneapolis, Minnesota

## Abstract

Advanced practitioners (APs) specializing in cancer care will most likely need to perform or participate in obtaining the history and physical (H & P) of a new patient. The core infrastructure of the history-taking and physical examination process remains the same across all patients regardless of diagnosis. There are, however, important distinctions in the H & P of the patient with cancer. These distinctions can be challenging for the student or novice oncology AP, leading to frustration and potentially poor patient satisfaction and outcomes. In each component of the patient history, certain considerations related to the cancer and its diagnosis and/or treatment to date must be included; these elements are different from those in the general medical H & P. This article focuses mainly on the structure and elements of the history of the present illness phase of the H & P. The similarities and differences between taking a cancer-focused H & P vs. a traditional medical one are discussed as well.

The oncology advanced practitioner (AP) needs to be well versed in the basics of a new patient history and physical (H & P). Most graduates of nurse practitioner and physician assistant programs feel at least moderately comfortable working through the traditional components of a new patient H & P, performing the formal presentation, and providing the appropriate documentation (Rosenzweig et al., 2012). The AP working in cancer care is faced with additional challenges. These foundational skills are critical, as the oncology AP must incorporate the traditional new patient H & P into the cancer-focused H & P to meet the needs of the patient with cancer.

In cancer care, the AP does not complete this assessment independently. The physician will ultimately confirm the diagnosis, perform the staging, and make the treatment decisions for someone newly diagnosed with cancer. In order to be a well-integrated member of the cancer care treatment team, the AP must understand the components of the new patient visit and the complex staging process that is necessary to determine the appropriate treatment and fully understand the likely disease course. Additionally, in prospectively assessing the complexities of each patient, the tumor characteristics, and the likely therapy, the AP can begin to involve the other health-care professionals from the cancer care team to facilitate a multidisciplinary, holistic plan of care (Fennell, Das, Clauser, Petrelli, & Salner, 2010).

## Five Key Steps

**Figure 1:**
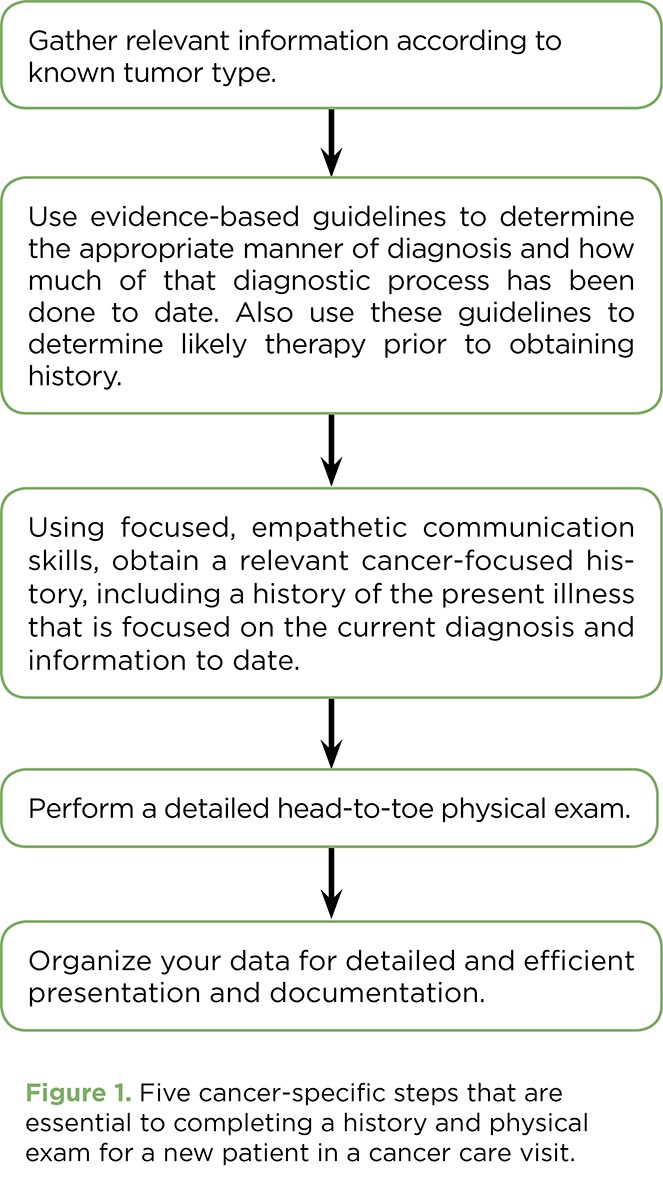
Five cancer-specific steps that are essential to completing a history and physical exam for a new patient in a cancer care visit.

The reader is referred to traditional "gold standard" sources such as the *Bates’ Guide to Physical Examination and History Taking* for obtaining and recording the foundational H & P exam (Bickley & Szilagyi, 2012). There are five cancer-specific steps that are essential to completing the H & P for a new patient in a cancer care visit: (1) gathering information according to tumor type, (2) determining the appropriate manner of diagnosis for that tumor type and how much of that diagnostic process has been done to date, (3) obtaining a relevant cancer-focused history, (4) performing the physical exam, and (5) performing appropriate organization of the data to make a professional presentation and complete appropriate documentation (see Figure 1).

These five steps must be followed when a patient is newly diagnosed, newly referred to a cancer specialty, or new to the practice with a history of cancer. In order to bill at a high level of complexity—the level of billing most appropriate to cancer care for new patients with cancer—the components of the visit and the documentation must reflect complex medical decision-making. Additionally, the patient must truly meet the Medicare definition for a new visit: "A new patient is: A patient who has not received any professional services, i.e., E/M service or other face-to-face service (e.g., surgical procedure), from the physician or physician group practice (same physician specialty) within the previous 3 years." (Centers for Medicare & Medicaid Services [CMS], 2014).

## Preparation

A new patient can appear in a variety of cancer care settings; collating information from previous providers can be challenging. For outpatients or planned inpatient admissions, the gathering of background information is ideally performed prior to the visit in the event that the search for this information is particularly time consuming or complicated. Having a sense of the patient complexity, eligibility for clinical trials, possible comorbidities, educational requirements, and need for psychosocial support is also helpful for daily resource (time, clinical staff) planning for both the AP and other collaborative cancer care professionals (Fennell et al., 2010). Each tumor type will have specific criteria for what is needed for treatment decisions to be made.

## Importance of National Guidelines

In order to prepare for a new patient evaluation in cancer care, the AP must first know the specific type of cancer for which the patient is being evaluated or treated. Once the cancer diagnosis is established, the AP can refer to evidence-based national guidelines such as those put forth by the National Comprehensive Cancer Network (NCCN) to gather the information needed for the complete staging of that specific cancer or tumor type (Winn & McClure, 2003). It is not enough simply to know the traditional tumor–lymph node–metastasis (TNM) staging for a new patient visit. In today’s cancer care environment, treatment decisions are based on a multitude of factors. These include the traditional consideration of tumor size, the specific lymph node evaluation, and the radiology performed for possible metastatic disease. However, treatment decisions extend beyond these traditional parameters. Considerations also include the specific molecular or genetic markers necessary to determine both prognostic (the natural history of the tumor) and predictive (effective therapies) information (Weigel & Dowsett, 2010).

The evidence-based guidelines provide the appropriate diagnostic workup that needs to be completed. This is a complex but important process. When all cancer clinicians follow these national guidelines, it ensures a consistent state-of-the-art diagnostic approach that is the basis for the most tailored and appropriate therapy. Guideline utilization can also help to mitigate geographic or socioeconomic disparity in cancer care (Smith & Hillner, 2001; Eddy, 2005).

## Pathology Review

The NCCN Guidelines are nationally recognized standards for clinical practice screening, diagnosis, and treatment of cancer. As mentioned previously, once the patient’s tumor type is established the AP can search the guidelines for information on the necessary testing needed for complete staging and information. From these guidelines, the AP can create a checklist of the items needed for complete diagnostic information and assess what is available in (and missing from)the existing medical record.

These guidelines are dense, and they can be difficult for the novice to navigate. First, the appropriate type of surgery (and timing) that is necessary for a specific cancer is detailed. Next, the appropriate diagnostic procedures, bloodwork, and radiology necessary for staging each specific tumor are listed. The pathology evaluation can be particularly challenging. The AP needs to follow the footnotes in the guidelines for the principles of pathologic review (NCCN, 2014). These principles will differ according to tumor type.

The details of the appropriate tumor and lymph node evaluation must be noted. The appropriate investigation of lymph nodes, including the total number of nodes to be examined, is different for each malignancy. The AP can then compare the tumor and lymph node evaluation that was completed and available in the patient’s existing record against these standards. Specific biologic markers and genetic mutations considered clinically useful for prognosis or predictive of response to specific therapy for that tumor will also be listed. The characteristics of the tumor that need to be noted are not just size but may also include grade or degree of tumor differentiation and/or markers indicating the pace of cellular division as per specific tumor type (Vogel, 2010).

The issue of any genomic or proteomic information relevant to clinical decision making is always evolving, challenging APs to understand the continually expanded knowledge regarding carcinogenesis and pathways of cellular activation that are targets for therapy (Ludwig & Weinstein, 2005). Measurement issues of the proteomic evaluation can be controversial, and the AP should be aware of "gold standard" measurement for commonly utilized proteomic information for each tumor type. These results then should be included in the patient history or noted that they should be obtained.

**Figure 2:**
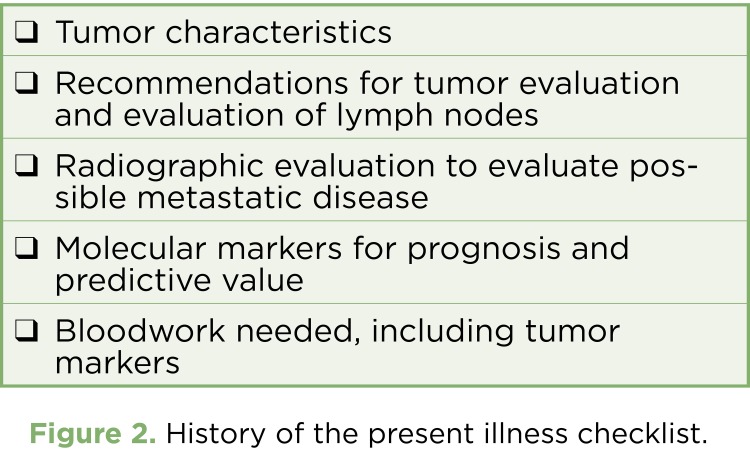
History of the present illness checklist.

From these guidelines, the AP will have a checklist of what needs to be found in the patient record to have complete and appropriate staging for the specific malignancy. Although this sounds quite complex, these checklists can be used routinely for commonly encountered tumors; this can make the preliminary work much less cumbersome and time consuming (see Figure 2). Additionally, the checklist or reminder sheet can be used after the patient is seen to prepare for the oral patient presentation or for documentation. If key information is missing, it may need to be obtained from the appropriate source or outside institutions, ideally prior to the patient visit.

Prior to meeting the patient, the AP should again use the national guidelines to determine the most likely therapy for this specific patient. If possible, it is helpful to discuss the likely treatment with the physician prior to seeing the patient so the cancer care team portrays itself as consistent and able to communicate among its members (Fennell et al., 2010). That is important because the history will then be focused on determining the likelihood that this specific patient can physically and emotionally withstand the projected therapy.

## History of the Present Illness

The critical piece of the patient’s history that helps to clarify the "story" as it relates to the presenting complaint is the history of the present illness (HPI). In the traditional medical exam, the HPI is an opportunity to learn about the characteristics of the presenting sign or symptom. The onset, location, duration, characteristics, aggravating and relieving factors, and temporality of the presenting sign or symptom are noted (Bickley & Szilagyi, ). The HPI in cancer care, specifically medical oncology, has a different focus in that the diagnosis is usually well established; it is not a diagnostic mystery. What is required in medical oncology is the establishment of the "story" of the cancer diagnosis; the diagnostic workup to date; and any relevant social, family, or past medical history that has an impact on the cancer diagnosis and potential treatment. Additionally, the HPI in cancer provides an important opportunity for the patient to retell a story that is emotionally difficult, starting to put the emotional trauma of cancer diagnosis into some perspective (Adler, 1997).

Core therapeutic competencies that should be present in any patient interview are respect, genuineness, and empathy. These qualities should be integrated into the interaction and are more important to patient outcomes than the actual verbiage of the discussion (Coulehan & Block, 2006). With an empathetic listener, the retelling of a medical trauma can itself be therapeutic (Cepeda et al., 2008). This exchange also provides the AP obtaining the history with an opportunity to establish a bond with a new patient and his or her family members and to assess the patient’s emotional response to the cancer diagnostic process. The retelling of the patient’s experience will also provide some insight into the patient and family’s competence in dealing with the health-care system and their level of resilience and potential for distress during further cancer therapy.

The components of the HPI in cancer care should begin with "the patient was in his [or her] usual state of health when…" thus beginning the story of the cancer diagnosis, or what is known to date. Usually a patient will relay a story of noting an abnormality in a sign or symptom, sometimes self-treated or disregarded as not serious for some time, which was ultimately presented to a medical professional. Delays in obtaining routine screening or in seeking evaluation of a sign or symptom that results in a cancer diagnosis, for whatever reason, can result in patient guilt: They can feel that they "brought it on themselves." The oncology professional should look for ways to utilize empathetic statements by recognizing and responding to the emotion (McCormack et al., 2011). If the AP detects self-blame, an empathetic response might be an acknowledgment that many patients feel this way, with a statement about focusing on what is best moving forward.

It is unusual for patients presenting with signs or symptoms of cancer to see a cancer care provider first. A helpful way to obtain and present the HPI for a patient with cancer can be through a timeline rather than as signs or symptoms that require diagnosis (Skeff, 2014). For example, patients usually first present to a primary care physician or a host of specialists who may analyze the signs and symptoms and begin the diagnostic workup, with cancer in the differential diagnosis. The providers will order radiology, lab work, and/or biopsy, resulting in a cancer diagnosis. Depending on the tumor type, the patient may then go to surgery or come directly to medical oncology. It is important to capture the providers and the timeline in the medical oncology patient’s HPI.

One common error made by new APs in cancer care is focusing on the diagnostic process if there was a delay in establishing the diagnosis. For example, if a patient ultimately diagnosed with ovarian cancer presented to several providers with abdominal pain and discomfort without an immediate diagnosis, the delay is important to note briefly, but the AP should not go into explicit detail about every provider and every visit in the documentation and verbal presentation. That is important background information, but unlike a medical history, when the diagnosis is not known, this information is no longer particularly relevant. The AP in cancer care can certainly garner the emotional impact of the diagnostic delay on the patient, but the important information from this point forward is the cancer evaluation. Once the timeline is established, the oncology NP can then gently ascertain if the patient and family know why they were referred to this cancer care provider and, if possible, begin to manage uncertainty by determining the patient’s thoughts about the possibility of cancer therapy (McCormack et al., 2011).

The creation of the HPI in cancer care is a unique opportunity to gather all of the relevant information regarding the cancer diagnosis for the treatment decision to be made with the best information. The HPI allows the AP to place that information in the context of the patient’s story and to begin to assist the patient and family with the creation of an illness narrative (Donnelly, 1988).

Once the cancer story is established, the rest of the history is then completed in the manner of a traditional history, with an eye toward any important information being moved to the HPI. Components of the past medical history, social history, family history, or review of systems may or may not be relevant to the presenting cancer and the ability to appropriately treat with systemic therapy. The decision that the AP will make to move components of the patient’s history to the HPI is based on the impact that this information would have on prognosis or the ability of the patient to receive cancer therapy without great physical or emotional distress.

## Other Components of the History

**Past Medical History**

The past medical and surgical history is usually completed in accordance with standard history-taking methods. All medical illnesses need to be documented with length of illness, current treatment, provider following the illness, long-term sequelae, and frequency and type of follow-up visits (Bickley & Szilagyi, 2012). The past medical history should include a list of medications, including nutritional and medicinal supplements and complementary therapies the patient has chosen (Bickley & Szilagyi, 2012). It is interesting to question the patient and family about the reason certain supplements were chosen, their knowledge regarding the supplement, and their belief system regarding supplementary or complementary therapies. The past surgical history is completed as per routine evaluation.

**Family History**

The family history in cancer takes a bit of a different focus than a traditional medical history. In a traditional medical history, the focus is on risk and determination of likely disease. In cancer care the family history of cancer needs to be explored at great length (Lu et al., 2014). Primary (mother, father, and siblings) and secondary (cousins, aunts, uncles, and grandparents) histories of family cancer diagnosis must be established. If there is a family history of cancer noted, the AP should determine the age at diagnosis, the course of the illness, and the patient’s reaction to the illness. This is done not only to determine a patient’s cancer risk but also as an indication for further genetic evaluation in order to determine future patient and/or family risk (Lu et al., 2014). The other illnesses or causes of death that are present in the patient’s family history should be noted, as in the traditional medical history, but this is not usually critical information for the cancer care provider.

**Patient Profile**

In cancer care, the patient profile or the social history also takes on a great deal of importance. The traditional questions including alcohol, tobacco, and recreational drug use are included. A slight difference in cancer care might be the self-blame that patients can have regarding their lifestyle choices. A patient newly diagnosed with lung cancer will probably have some emotional reaction to a question about tobacco use. An empathetic response, rather than any response that may reflect judgment, is most effective. The response from the AP could reflect the emotional difficulty the patient is encountering. 

In addition to the traditional questions of the social history, the cancer social history should include details of the patient’s lifestyle that may impact the ability to receive the recommended cancer therapy. Having the patient describe a typical day is particularly helpful in understanding the potential impact of cancer treatment (Coulehan & Block, 2006). Other key issues in the patient profile include distance from home to the cancer center, employment, family responsibilities, social support, and economic problems including housing, food stability, transportation, and the potential burden of incidental costs associated with cancer therapy. The AP will not be able to "solve" all of these issues, but they should be acknowledged during the discussion. These social issues can represent barriers to receiving full appropriate therapies, particularly if the therapies include a clinical trial or a complex regimen. This information can also trigger a proactive social services referral, a valuable resource in mitigating cancer-related distress during therapy (Zebrack, Burg, & Vaitones, 2012).

**Review of Systems**

The review of systems, the last portion of the patient history, can be used for several purposes during the initial patient visit in cancer care. There are usually institutionally approved review of systems checklists that will be completed by the patient in order to meet the Center for Medicare and Medicaid criteria 14-point review (CMS, 2010). The AP can review the patient’s checklist and have him or her elaborate upon the positive responses so the sometimes tedious "laundry list" of symptoms does not have to be asked and answered in person.

In cancer care, the focus of the review of systems should be on signs and symptoms that may result from the cancer, the diagnostic treatment thus far, and the anxiety and worry surrounding the diagnostic process. Tumor-related considerations to be reviewed include pain, performance status, weight, appetite, bowel and bladder symptoms, and specific questions related to likely symptoms associated with certain cancers (abdominal bloating with ovarian cancer, shortness of breath with lung cancer). These need to be reviewed. In addition, the AP should query the patient and family members regarding the emotional toll of the newly diagnosed cancer. Anxiety, sleep disturbance, and emotional lability are common in patients with recently diagnosed cancer (Whelan et al., 1997) and should be assessed.

**Physical Exam**

The physical exam is extremely important to the new patient visit. Although several physical exams may have been completed during the diagnostic process, the AP should complete and document a thorough head-to-toe exam. This is important because it will serve as a baseline physical exam as the patient embarks on cancer therapy. Additionally, even a subtle abnormal physical exam finding may trigger further evaluation for metastatic disease, completely changing the staging, subsequent treatment, and prognosis.

**Summary of Information**

Once the H & P is complete, the AP must collate information and present this patient information in oral and written documentation. The notes and/or checklist used for the history preparation will assist with the oral and written presentations. The attending physician should review the radiology and pathology reports, which should be readily available in electronic or hard copy form so that the flow of the presentation is not interrupted. In a presentation, the AP should include all of the pertinent positives and negatives from the other components of the history to present a picture of the cancer diagnostic story in the context of the whole patient.

## Conclusion

The taking of an initial history from the patient with cancer and his or her family members is challenging, and there are some important differences as compared with the standard medical history. The incorporation of components specific to cancer requires a commitment to flexibility, ongoing education, and the ability to gather complex information while establishing an empathetic relationship. The process becomes easier as the AP garners experience with different tumor types, receives constructive feedback, and grows more confident. 
